# Diffuse stimulant-associated vasculopathy presenting with acute limb ischemia, compartment syndrome, and multifocal visceral infarction

**DOI:** 10.1093/jscr/rjag930

**Published:** 2026-10-19

**Authors:** Syed Azaan Adnan, Julianne Wanner, Syed Moaiz Adnan, Christopher Thompson

**Affiliations:** Campbell University School of Osteopathic Medicine, Leon Levine Hall of Medical Sciences, 4350 US Hwy 421 S, Lillington, NC 27546, United States; Campbell University School of Osteopathic Medicine, Leon Levine Hall of Medical Sciences, 4350 US Hwy 421 S, Lillington, NC 27546, United States; Brock University, Department of Health Sciences, 1812 Sir Isaac Brock Way, St. Catharines L2S 3A1, Canada; WakeMed Health and Hospitals, Department of Addiction Medicine, 3000 New Bern Ave, Raleigh, NC 27610, United States

**Keywords:** acute limb ischemia, compartment syndrome, cocaine, stimulant-associated vasculopathy, rhabdomyolysis, multisystem disease

## Abstract

Stimulant-induced vasculopathy typically presents as localized ischemia secondary to vasospasm and thrombosis. Diffuse involvement of multiple vascular territories is uncommon and may result in severe, multisystem complications and increased morbidity. We report a 40-year-old female with polysubstance exposure, including cocaine and amphetamines, who presented with acute right lower extremity ischemia and compartment syndrome. Computed tomographic angiography demonstrated distal tibial, peroneal, and posterior tibial arterial occlusion. The patient underwent emergent four-compartment fasciotomy, allowing Doppler signals to return. Postoperative complications included rhabdomyolysis, acute kidney injury, bilateral adrenal infarctions, left renal infarction, and persistent foot drop. This case highlights the need to consider stimulant-associated vasculopathy in patients with acute limb ischemia without an embolic source and to evaluate for concurrent renal and visceral ischemic injuries. Early recognition and multidisciplinary management are essential to reduce morbidity.

## Introduction

Acute limb ischemia is a vascular emergency requiring rapid diagnosis and intervention to prevent limb loss, systemic complications, cardiovascular morbidity, and mortality [1]. Although embolism or *in situ* thrombosis are the most common causes, stimulant-associated vasculopathy is also an important nonatherosclerotic etiology [2]. Amphetamines and cocaine have been well documented to cause intense arterial vasoconstriction along with thrombosis and end-organ ischemia through sympathomimetic effects [3]. Despite this association, widespread involvement of multiple vascular territories remains rare.

In this report, we present the case of a 40-year-old patient with polysubstance exposure who developed acute right lower extremity ischemia complicated by compartment syndrome, severe rhabdomyolysis, acute kidney injury (AKI), and multifocal visceral infarctions with left renal and bilateral adrenal involvement. This case illustrates the potential for stimulant-associated vasculopathy to cause extensive, multisystem ischemic injury and emphasizes the importance of maintaining a high index of suspicion for systemic complications in patients presenting with acute limb ischemia in the setting of substance use [4].

## Case report

A 40-year-old female with a history of chronic pain syndrome, hepatitis C, depression, and polysubstance use was brought to the emergency department with severe right lower extremity pain after being found on the floor ~36 h after a fall. On initial examination, the right lower extremity was pulseless, edematous, tense, and bullous with a mottled appearance (Fig. 1). All nerve distributions of the foot demonstrated diminished sensation and absence of motor function.

**Figure 1:**
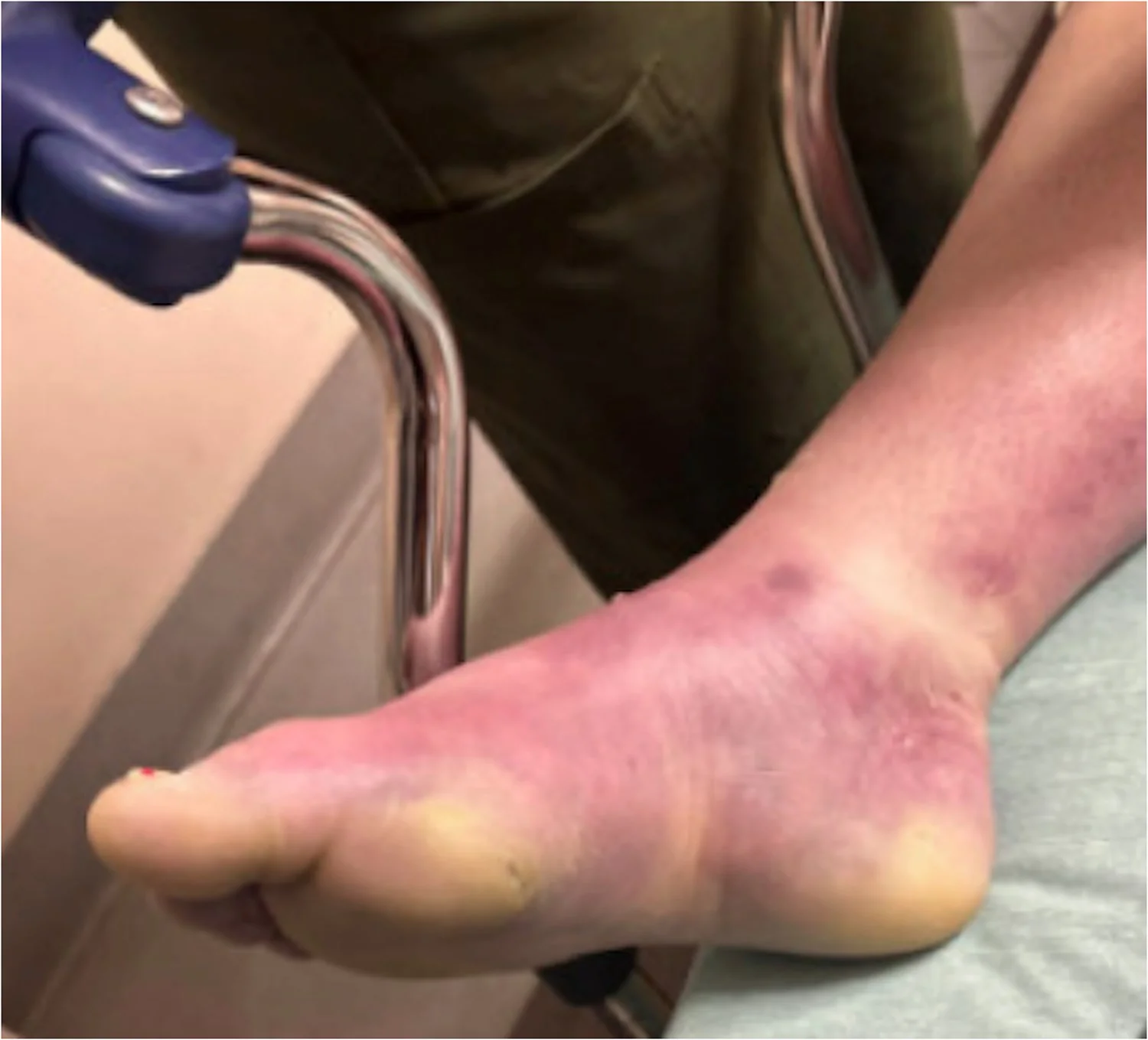
Gross appearance of right lower extremity at presentation; the extremity was edematous, tense, bullous, and mottled, consistent with acute compartment syndrome.

Urine toxicology was positive for cocaine, fentanyl, benzodiazepines, and amphetamines. Computed tomographic angiography (CTA) of the right lower extremity revealed occlusion of anterior tibial, posterior tibial, and peroneal arteries distal to the trifurcation (Fig. 2). Given findings consistent with Rutherford class IIb acute limb ischemia with worsening compartment syndrome, a heparin infusion was initiated and the patient emergently underwent a four-compartment fasciotomy. Emergent decompression was performed to relieve compartment pressure and prevent irreversible neuromuscular injury. No thromboembolus was identified intraoperatively as evidenced by two passes of a balloon embolectomy catheter without clot extraction. Intraoperatively, return of dorsalis pedis pulse was noted following fasciotomy. Postoperatively, restoration of blood flow was confirmed via Doppler detection of dorsalis pedis and posterior tibial pulses. Additional angiography was deferred postoperatively in the setting of AKI. Given the lack of intraoperative evidence of thromboembolus and return of distal flow following fasciotomy, the limb ischemia was suspected to be secondary to extrinsic arterial compression as opposed to embolus. Initial and postoperative laboratory studies were indicative of rhabdomyolysis secondary to compartment syndrome, with creatine kinase peaking on postoperative Day 1 at 65 750 U/l.

**Figure 2:**
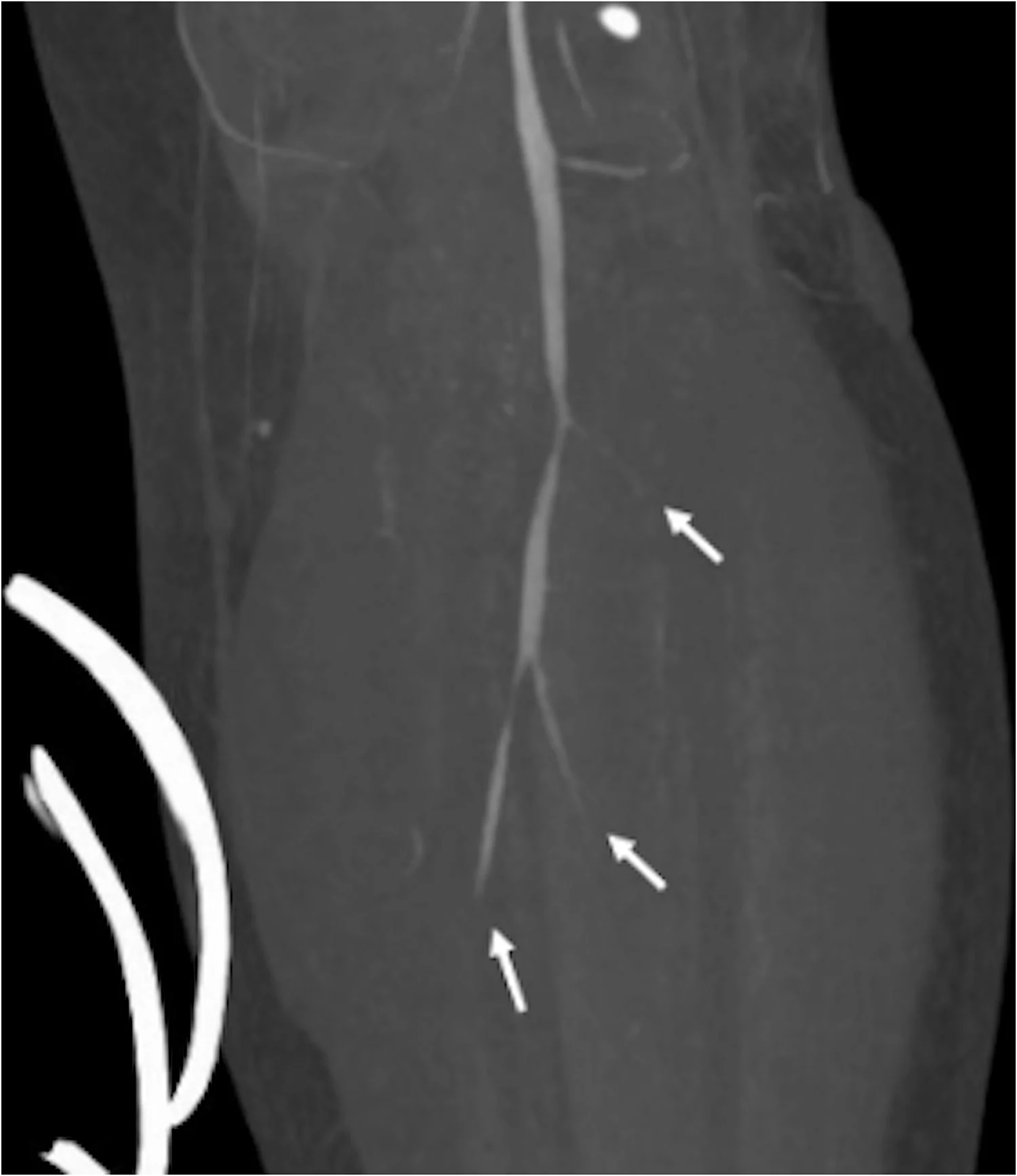
CTA of right lower extremity; imaging demonstrated occlusion of the anterior tibial, posterior tibial, and peroneal arteries distal to the trifurcation, with absent distal runoff.

Abdominal computed tomography on admission was notable for bilateral adrenal infarction and left renal infarction. The patient’s renal function continued to decline through postoperative Day 5, with serum creatinine peaking at 3.94 mg/dl. The etiology of AKI was suspected to be compounded pigment nephropathy from rhabdomyolysis, renal infarction related to cocaine use, reperfusion injury, and intravenous contrast exposure. Serum creatinine ultimately returned to normal range on postoperative Day 13.

Pain control and withdrawal management required multidisciplinary coordination between addiction medicine and hospitalist teams during the hospital course. The patient was discharged home with persistent right foot drop. Outpatient follow-up at a specialized primary care clinic was arranged.

## Discussion

This case illustrates a rare systemic presentation of stimulant-associated vasculopathy with simultaneous limb-threatening ischemia and multifocal visceral infarction. The absence of an intraoperative thromboembolus, distal distribution of arterial occlusion, concurrent visceral infarctions, and positive stimulant toxicology favored a stimulant-associated vasospastic-thrombotic process, although complete exclusion of alternative embolic or inflammatory causes was limited by the emergent presentation. Cocaine and amphetamines may cause vasoconstriction, endothelial dysfunction, platelet activation, and thrombosis, producing both macro- and microvascular ischemia [3, 5]. The presenting symptoms and history of this patient support a vasospastic-thrombotic process, although prolonged immobilization and compression secondary to prolonged down time also likely contributed to the development of a unilateral compartment syndrome and rhabdomyolysis [1].

A key feature of this case is that distal Doppler signals returned post-fasciotomy, suggesting that decompression can be limb-salvaging even when no thromboembolus is removed. Furthermore, combining vasospastic and prothrombotic properties assists in explaining simultaneous limb ischemia and systemic end-organ damage. In this case, occlusions without an identified intraoperative embolus favor an *in situ* or a mixed vasospastic-thrombotic process, although emergent limb salvage precluded a complete embolic workup at presentation. Diagnostic workup can best be used as exclusionary in nature. Improving patient outcomes with stimulant-associated acute limb ischemia at presentation would benefit from low-threshold engagement of standard embolic evaluation, and exclusion of common embolic and systemic causes of acute limb ischemia such as arrhythmia, intracardiac thrombus, proximal arterial pathology, antiphospholipid syndrome, vasculitis, and infectious emboli [2].

Prolonged ischemia further precipitated compartment syndrome [6] and rhabdomyolysis [7] with subsequent pigment-induced nephropathy, electrolyte derangements, and a residual neurologic deficit clinically represented as persistent right foot drop at discharge. AKI was likely multifactorial, reflecting pigment nephropathy from rhabdomyolysis, renal infarction, possible reperfusion injury, and contrast exposure [8]. Management priorities should emphasize urgent restoration of perfusion when indicated and fasciotomy for compartment syndrome, aggressive isotonic fluid administration, close electrolyte and urine output monitoring, and renal replacement therapy [2, 7, 9].

In this case, emergent limb decompression took priority, making the exclusion of a cardioembolic or hypercoagulable source limited as a complete post-operative embolic-source evaluation was not performed. Additionally, polysubstance exposure and prolonged immobilization limit attribution to cocaine alone. The presence of unilateral limb ischemia raises the possibility of prolonged immobilization in a sustained, nonphysiologic position contributing to the development of compartment syndrome. Hence, acute limb ischemia in patients with known or suspected stimulant use should prompt consideration of multifactorial vasculopathy even in the absence of an embolic source. Clinicians should maintain vigilance for systemic ischemic complications, as visceral infarctions may occur concurrently and may initially be clinically silent [10].

In conclusion, prompt surgical decompression resulted in the restoration of perfusion, with a residual neuromuscular deficit likely due to the timing of intervention relative to the initial vascular insult. Clinicians should consider stimulant-associated multifactorial vascular pathology in patients presenting with acute limb ischemia without an identifiable embolic source. Early evaluation for visceral ischemia and renal injury is critical for reducing morbidity, improving prognosis, and preventing limb loss.
